# Modified “C” Haptic Intraocular Lens Orientation and Negative Dysphotopsia

**DOI:** 10.3390/diagnostics14121288

**Published:** 2024-06-18

**Authors:** Valeria Cuevas-Lozano, Rosario Gulias-Cañizo, Oscar Guerrero-Berger

**Affiliations:** 1 Department of Anterior Segment Surgery, Fundación Hospital Nuestra Señora de la Luz, Mexico City 06030, Mexico; 2 Centro de Investigación en Ciencias de la Salud, Universidad Anahuac, Mexico City 01840, Mexico; 3 Centro Oftalmológico Mira, Mexico City 06760, Mexico

**Keywords:** dysphotopsia, negative dysphotopsia, cataract surgery, phacoemulsification, haptic, haptic orientation

## Abstract

Phacoemulsification is the standard of care in cataract surgery in the developed world, with patients having high expectations regarding visual results. Postoperative dissatisfaction due to negative dysphotopsia (ND) ranges from rare to very frequent; its etiology is unclear, and it affects postoperative satisfaction. Since one of the most frequently used strategies to avoid ND is related to intraocular lens (IOL) haptic orientation, we conducted a prospective interventional study that enrolled 197 patients who underwent standard phacoemulsification. All patients had a one-piece hydrophobic acrylic IOL implanted; in one group, the haptics were placed in any meridional axis except inferotemporal (IT) meridians, and in the other group, the IOL was implanted with the haptics in an IT position. Our results showed no statistically significant differences between groups when analyzing the correlation between the position of IOL haptics and the presence of ND in week one and month one. Also, pupillary diameter showed no statistically significant differences between patients with or without ND. Despite some studies claiming that haptic orientation prevents ND, we found that haptic orientation does not correlate with ND incidence and that ND decreases from day 1 to month 1. Our results support previous findings on the decrease in ND over time and that haptic orientation should not be considered an intraoperative strategy to avoid this unwanted phenomenon.

## 1. Introduction

Cataracts are one of the leading causes of reversible blindness worldwide, and phacoemulsification is considered the standard of care for cataract removal in the developed world. Phacoemulsification is a highly safe procedure with excellent visual outcomes, so postoperative dissatisfaction after uncomplicated phacoemulsification with in-the-bag IOL implantation is uncommon. Also, surgical advances and new intraocular lens (IOL) technologies have given patients high expectations regarding visual results. However, there is a relevant percentage of patients who report subjective complaints like pseudophakic dysphotopsia. Dysphotopsia is an unwanted optical phenomenon that may affect a patient’s quality of life and visual satisfaction, with rare cases that can cause visual disability. Dysphotopsia can be positive or negative, with a reported overall incidence that ranges from rare to 78% [1]. Positive dysphotopsias are bright artifacts described as arcs, starbursts, halos, or streaks, usually transient and that disappear spontaneously [1].

On the contrary, negative dysphotopsias (ND) are reported by patients as a dark shadow in the far temporal field, and their etiology is unclear. One theory states that the IOL does not focus light at considerable visual angles because it is much smaller than the natural crystalline lens it replaces [2,3]. Ray-tracing optical modeling, especially Holladay’s extensive work in this field, proposed that there is “a shadow when there is a gap between the retinal images formed by rays missing the optic of the IOL and rays refracted by the IOL” [4]. An experimental model in phakic eyes that simulates what happens in pseudophakic eyes with an annular opaque contact lens supports the hypothesis of light obstruction from the far temporal field [5].

The incidence of ND alone is up to 26% [6], and although in most cases it is temporary, around 1.5 to 3% have persistent ND [7,8], affecting postoperative satisfaction. The etiology of ND is now better understood; several bench and clinical studies have proposed potential causes, like temporal corneal incisions [9,10], a small pupil diameter [11], or increased angle kappa [11,12]. It has been reported that IOL tilt and decentration do not cause dysphotopsia [13], and most authors agree that ND is invariably associated with in-the-bag IOLs [14]. Table 1 includes primary and secondary factors known to increase the risk of ND, as reported by Holladay and Simpson [4].

There have been several strategies reported for the treatment of negative dysphotopsia, like intraocular lens optic truncation [15], sulcus-fixated IOL implantation [12], dedicated IOLs designed to treat ND [12,16], or ring implantation [17].

Other efforts have focused on IOL design, with some claiming to reduce the incidence of dysphotopsia, like the 7.0 mm optic diameter [18] or plate haptics [19] IOLs. One of the theories is that ND appears exclusively in IOLs with an overlapping anterior capsule, so removal of the nasal anterior capsule remnant with neodymium:YAG (Nd:YAG) laser has been advocated as a successful treatment for chronic ND [20,21].

However, one of the most frequently used strategies to avoid ND is related to IOL haptic orientation. There has been much controversy regarding the haptic position that may prevent ND. Some reports state that acrylic IOLs with an inferotemporal orientation resulted in a 2.3-fold decrease in the incidence of ND [22], while other reports support the horizontal position [23,24]. Since haptic orientation is one of the main factors considered as causative of ND, we conducted a clinical study to evaluate the correlation between haptic orientation and ND in a tertiary eye care institution.

## 2. Material and Methods

This prospective study was performed at a tertiary eye care center, adhered to the latest tenets of the Declaration of Helsinki, and was approved by the Ethics Committee of the Fundación Hospital Nuestra Señora de la Luz (approval code 2022S7R3). We screened consecutively all patients >50 and <99 years of age eligible for routine phacoemulsification until we reached the sample size for the study, a total of 197 eyes from 197 patients. Exclusion criteria included age < 50 years and >99 years of age, a diagnosis of diabetes mellitus, pseudoexfoliation, glaucoma, previous intraocular surgery, previous ocular trauma, corneal leucoma, or any macular or optic nerve disorder affecting visual function. All patients provided informed consent for study participation. Two experienced surgeons performed all surgeries, consisting of routine standard phacoemulsification. Routine cataract surgery in our department is performed as follows. After immediate preoperative antisepsis of the periocular skin, eyelids, and ocular surface with 5% povidone-iodine using local anesthesia with proparacaine eye drops, we make a 2.4 mm clear corneal incision followed by ophthalmic viscoelastic injection to proceed with capsulorrhexis. Afterward, hydrodissection and nuclear rotation are followed by phacoemulsification (Centurion^®^ Vision System, Alcon Laboratories, Fort Worth, USA), cortex aspiration, and IOL implantation (enVista^®^ MX60; Bausch and Lomb Incorporated, Rochester, NY, USA). After implanting the IOL, we perform thorough viscoelastic aspiration and suture the phaco incision with 10-0 nylon.

Regarding IOL orientation for this study, patients were randomly assigned to two groups. All patients had a one-piece hydrophobic acrylic IOL implanted, as previously mentioned. The enVista MX60 is a monofocal IOL characterized by a neutral aspheric, aberration-free optic, and scratch-resistant material, with a sharp square edge, posterior-vaulted haptics, and a refractive index of 1.54. For the two study groups, patients were divided as follows. The IOL was implanted with the haptics in the meridional axis selected by each surgeon (excepting inferotemporal (IT) meridians) in one group, and the same IOL with the haptics placed in an IT position in the second group. The definition of IT orientation in the right eye was positioning the haptics on the 7–8 o’clock meridians. In the left eye, the orientation considered as IT was at 4–5 o’clock meridians. After surgery, we evaluated the presence of dysphotopsia at one week and one month using a question validated by a previous study [23]: “Do you perceive a dark or grey shadow to the side of your vision?” The patient and the researcher asking the question were blinded regarding haptic orientation. Finally, we confirmed the position of the haptics at the same time points by slit-lamp examination under pharmacologically induced mydriasis. Since small pupil diameter has been considered a potential cause of ND, we included a random subgroup of 47 eyes with pupil diameter measurements. Pupil diameter data were analyzed only at the 1-month timepoint since there are no expected pupil size variations from one week to one month without the occurrence of trauma, surgery, or a neurological disorder.

## 3. Statistical Analysis

Data were captured in an Excel spreadsheet (Microsoft^®^ Excel^®^ for Microsoft 365 MSO, version 2404) without identifying patient data and transferred to an SPSS file (SPSS Statistics, IBM) for statistical analysis. Numerical variables were described with central tendency and dispersion measures, and categorical variables with absolute numbers and percentages. The correlation between categorical variables was evaluated using the Chi-square test and McNemar test when applicable. The relationship between numerical and categorical variables was analyzed with the student’s T test or ANOVA for two or more groups, respectively.

## 4. Results

Overall, we included 197 eyes that fulfilled all inclusion criteria and none of the exclusion criteria. There were 64 eyes in the IT group, and 133 eyes in the other groups: 30 eyes in the Vertical position, 46 eyes in the Horizontal position, and 57 eyes in “Other”. Demographic data and laterality of the included eyes are shown in Table 2. The mean age was 67.63 years (SD 13.31), and there was a slight predominance of women versus men (56.3 vs. 43.7%). There were slightly more right than left eyes (53.8% vs. 46.2%). The proportion of eyes with IOL haptics at each specific position and the proportion of patients reporting ND at week one and month one are shown in Table 3. In the first week, most of the patients had haptics in the inferotemporal (IT) position, followed by other positions (O), horizontal (H), and vertical (V) positions (32.5, 28.9, 23.4, and 15.2%, respectively). At this time point, 45 (22.8%) patients reported ND. The order of frequency of haptics position was the same at one month, despite changes in absolute numbers and percentages (IT, O, H, and V; 35.5, 32.0, 22.3, and 10.2%, respectively). Also, the proportion of patients reporting ND was much lower at one month (22 patients, 11.2%) compared to week 1. The decrease in ND from week 1 to one month (22.8% vs. 11.2%) was statistically significant (*p* = ˂0.001). On the other hand, there was no statistical significance in haptic position changes between one week and one month (*p* = 0.362).

When analyzing the correlation between the position of IOL haptics and the presence of ND at week 1, there were no statistically significant differences (*p* = 0.514). Similarly, there was no statistically significant association between the position of IOL haptics and the presence of ND at one month (*p* = 0.361) (Table 3).

Finally, 47 patients had their pupillary diameter measured, so this variable was analyzed only within this group. The mean pupillary diameter of this group was 4.160 mm (SD 0.6167). There were no statistically significant differences in pupillary diameter when compared by gender (*p* = 0.949), eye laterality (*p* = 0.308), haptic position at one week (*p* = 0.638) or at one month (*p* = 0.694) (Table 4). Finally, when analyzing the distribution of pupillary diameter in patients with and without negative dysphotopsia, there were no statistically significant differences at one week (*p* = 0.077) or one month (*p* = 0.936).

Finally, there were no intraoperative or postoperative complications in any patients included in this study.

## 5. Discussion

Persistent ND has been treated successfully or reduced with procedures like anterior optic capture, sulcus IOL placement, and neodymium:yttrium-aluminum-garnet nasal capsulotomy, among others [12,15,16,20,21]. Studies have established several factors that influence the incidence of ND, like pupil size or the power or design of IOLs [4]. Since controlling all variables in a clinical study on this topic is not feasible, we decided to focus on a strategy that would not alter the refractive objective or surgical plan of phacoemulsification and, therefore, minimize any unnecessary risks derived from the study. We controlled intraoperative haptic orientation without modifying our standard phacoemulsification practice and evaluated its relationship with ND.

When deciding which orientation to choose and after reviewing the literature, we concluded that the most feasible hypothesis was that haptics in a superonasal position would induce a change in light refraction that could potentially reduce the gap proposed by Holladay [4]. Based on this, we decided to place the haptics in the IT position using only one type of IOL, comparing this to all other orientations.

Our results show that neither orientation decreased the incidence of ND with a follow-up duration of 4 weeks. This contrasts with a previous study that reported that IT orientation is a preventive strategy to reduce the incidence of ND [22]. However, this decrease was only observed in the immediate postoperative period and was no longer statistically significant after one week or one month, consistent with our study. Other studies concluded that the horizontal position better reduces the incidence of this phenomenon [23,24], but likewise, both studies had a short-term follow-up of 4 and 6 weeks, respectively.

A consistent observation in all studies is that ND appear following cataract surgery and decrease over time, leaving about one-fifth of the initially affected patients with permanent ND [25]. Our results also showed a higher incidence of ND in the first week vs. the first postoperative month, with a 50% decrease from week one to week four. We believe this is due to the expected postoperative peripheral capsular fibrosis that reduces capsular transparency and, therefore, the amount of light reflected in the peripheral nasal regions of the retina, thus reducing the gap reported by Holladay [4].

The weaknesses of this study are that although we had a pupil size subgroup, we did not consider other eye-dependent variables, such as axial length and anterior chamber depth, that could have helped classify the sample according to different anteroposterior eye diameters to evaluate a range from myopic to hyperopic eyes and perform an analysis that could detect differences between these groups. On the other hand, regarding the design of the intraocular lenses, we included only one aspheric, square-edged model, excluding the possibility of comparing whether other models with different asphericity or platforms would show different outcomes. At the same time, using a single IOL model is a strength of our study since it implies that the optical behavior is consistent in all patients. Another strength of this study is that we strictly controlled the recording of the IOL position, which was evaluated only by one blinded researcher, and under pupil dilation to confirm haptic orientation.

## 6. Conclusions

Our results support previous studies that reported that ND decreases over time after phacoemulsification and that haptic orientation is not a long-term prevention strategy for permanent ND. Additional studies are needed, including other variables such as axial length or angle kappa, and comparing different IOL designs, among other factors, to continue studying this complex postoperative phenomenon.

## Figures and Tables

**Table 1:** Factors that increase the risk of negative dysphotopsia (in order of importance).

Primary	Secondary
- Smaller photopic pupil - Larger positive angle kappa - Shape of IOL - Smaller axial distance of IOL behind iris - Nasal anterior capsule overlying anterior nasal IOL - Higher dioptric power if equi-biconvex or plano-convex - Optic–haptic junction of IOL not horizontal (or superonasal by 30°)	- Edge design (truncated versus rounded and thickness) - IOL material (higher versus lower index) - Negative aspheric surface(s)

IOL = intraocular lens. From Holladay and Simpson [4].

**Table 2:** Demographics and laterality.

		IT *n* = 64	Vertical *n* = 30	Horizontal *n* = 46	Other *n* = 57	*p*-Value **
Age *	67.63 ± 13.31	67.52 ± 13.58	69.80 ± 12.88	66.46 ± 13.75	67.58 ± 13.08	0.765
Gender ^ǂ^ Male Female	86 (43.7) 111 (56.3)	24 (37.5) 40 (62.5)	14 (46.7) 16 (53.3)	24 (52.2) 22 (47.8)	24 (42.1) 33 (57.9)	0.474
Laterality ^ǂ^ Right Eye Left Eye	106 (53.8) 91 (46.2)	34 (53.1) 30 (46.9)	19 (63.3) 11 (36.7)	23 (50.0) 23 (50.0)	30 (52.6) 27 (47.4)	0.704

* Result expressed as mean (standard deviation). ^ǂ^ Results expressed as absolute numbers (percentage). ** *p*-value for intergroup comparisons. IT: inferotemporal.

**Table 3:** Intraocular lens haptics orientation and negative dysphotopsia at one week and one month.

Outcome	IT	Vertical	Horizontal	Other	*p*-Value *
Haptic position (Week 1)	64 (32.5)	30 (15.2)	46 (23.4)	57 (28.9)	0.514
Negative dysphotopsia (Week 1)	14 (31.1)	5 (11.1)	14 (31.1)	12 (26.7)	
Haptic position (Month 1)	70 (35.5)	20 (10.2)	44 (22.3)	63 (32.0)	0.361
Negative dysphotopsia (Month 1)	10 (45.5)	0 (0)	5 (22.7)	7 (31.8)	

All results expressed as absolute numbers (percentages); * *p*-value for intergroup comparisons. IT: inferotemporal.

**Table 4:** Description of pupil diameter subgroup.

	Number of Eyes	Mean ± SD	*p*-Value
Male	24	4.138 ± 0.58	
Female	23	4.183 ± 0.65	*p* = 0.949
OD	18	4.033 ± 0.57	
OS	29	4.238 ± 0.63	*p* = 0.308
Week 1			
Inferotemporal	13	4.092 ± 0.71	
Vertical	6	4.383 ± 0.68	
Horizontal	9	4.278 ± 0.50	
Other	19	4.079 ± 0.59	*p* = 0.638
Week 4			
Inferotemporal	19	4.216 ± 0.68	
Vertical	8	4.138 ± 0.74	
Horizontal	8	4.288 ± 0.33	
Other	12	4.000 ± 0.60	*p* = 0.694

## Data Availability

The raw data supporting the conclusions of this article will be made available by the authors on request.
